# Impact of 4% Deltamethrin-Impregnated Dog Collars on the Incidence of Human Visceral Leishmaniasis: A Community Intervention Trial in Brazil

**DOI:** 10.3390/pathogens13020135

**Published:** 2024-02-01

**Authors:** Guilherme Loureiro Werneck, Fabiano Borges Figueiredo, Maria do Socorro Pires e Cruz

**Affiliations:** 1 Departament of Epidemiology, State University of Rio de Janeiro, Rio de Janeiro 20950-000, RJ, Brazil; 2 Instituto for Public Health Studies, Federal University of Rio de Janeiro, Rio de Janeiro 21941-630, RJ, Brazil; 3 Carlos Chagas Institute, Oswaldo Cruz Foundation, Curitiba 81350-010, PR, Brazil; fabiano.figueiredo@fiocruz.br; 4 Department of Veterinary Morphophisiology, Federal University of Piauí, Teresina 64049-550, PI, Brazil; mspcruz@ufpi.edu.br

**Keywords:** visceral leishmaniasis, impregnated collars, prevention and control, intervention studies

## Abstract

Background: In Brazil, human visceral leishmaniasis (HVL) is caused by the protozoan parasite *Leishmania infantum*, primarily transmitted by the sand fly *Lutzomyia longipalpis*, with dogs acting as the main urban reservoir. This study aims to evaluate the effectiveness of 4% deltamethrin-impregnated dog collars (DMC) on HVL incidence. Methods: This is a community intervention study carried out from 2012 to 2015 in the municipalities of Araguaína, State of Tocantins, and Montes Claros, State of Minas Gerais, Brazil. Two areas in each were randomly allocated to either (1) culling seropositive dogs + residual insecticide spraying (control area—CA) or (2) culling seropositive dogs + residual insecticide spraying + DMC fitted to dogs every six months for two years (intervention area—IA). Cases of HVL (n = 1202) occurring from 2008 to 2020 were identified from the Brazilian Reportable Diseases Information System and georeferenced to the control and intervention areas. The HVL cases from 2008 to 2012 were considered as occurring in the “pre-intervention” period. Those cases from 2013 to 2016 and from 2017 to 2020 were regarded as occurring in the “intervention” and “post-intervention” periods, respectively. We used a mixed-effects Poisson regression model to estimate the effectiveness of the intervention, comparing the changes from the pre-intervention period to the intervention and post-intervention periods in the control and intervention areas. Results: In Araguaína, there was a statistically significant reduction in the incidence of HVL in both the control and intervention areas, comparing both the intervention and post-intervention periods with the pre-intervention period. The intervention with DMC was significantly associated with a reduction in HVL when comparing the intervention and pre-intervention periods, yielding an effectiveness estimate of the DMC of 27% (IC95% 1–46%, *p* = 0.045). No differences were observed when comparing the pre- and post-intervention periods (*p* = 0.827). In Montes Claros, cases reduced in both the control and intervention areas from the pre-intervention period to the intervention period (*p* = 0.913). In the post-intervention period, the incidence increased in the control area, while cases continued to decrease in the DMC area (*p* = 0.188). Conclusions: The use of DMC was associated with a reduction of 27% in the incidence of HVL during the period of DMC delivery, indicating that DMC is effective as an additional strategy for controlling visceral leishmaniasis in Brazil. However, no significant reduction associated with DMC was detected after the intervention period, suggesting that a control program based on the large-scale deployment of DMC might have to be maintained for more extended periods without interruption.

## 1. Introduction

Human visceral leishmaniasis (HVL) is a severe disease caused by parasites of the genus *Leishmania*. In the Americas, the parasites are transmitted by phlebotomine sand flies of the genus *Lutzomyia*, and domestic dogs serve as the primary reservoir for the protozoan parasite *Leishmania infantum*, playing a role in amplifying infection and contributing to transmission to humans [1].

Controlling zoonotic HVL has been challenging for public health officials and researchers in Brazil. Alongside dengue, HVL control has been considered a significant failure in the control of communicable diseases in Brazil [2]. Since the implementation of the Brazilian Program for Surveillance and Control of Visceral Leishmaniasis (PSCVL) in the early 1960s, the disease has undergone a significant process of urbanization and geographical spread, and the epidemiological situation is far from showing any substantial progress [3].

In the early stages of the PSCVL launched in 1963, the main proposed control strategies were mandatory reporting, timely diagnosis and treatment of human cases, vector control with insecticides, and euthanasia of seropositive dogs. These strategies seemed appropriate for addressing a disease that was primarily rural, occurring in specific environments such as foothills. However, Brazilian society underwent significant changes from then on, with an inexorable urbanization process. Massive population movements were reported from the drought-affected rural areas in the country’s northeast region. Initially, migration was towards the outskirts of the capitals and major cities of this region and, later, to other regions of the country. This intense and rapid urbanization process led to social segregation, with the fringes of large metropolitan areas being characterized by a lack of urban services, environmental destruction, and poor living conditions. This was the ideal scenario for the introduction and maintenance of the zoonotic visceral leishmaniasis cycle, with domestic dogs as the main reservoir of infection and the primary vector, the sand fly *Lutzomyia longipalpis*, adapting well to the new peri-domiciliary environment [4].

However, all these transformations in Brazilian society and the epidemiology of visceral leishmaniasis were not accompanied by substantial modifications in the control strategies advocated by the original PSCVL. However, only some well-designed epidemiological studies support their extensive use, with most showing limited effectiveness and only in specific contexts [5]. New promising approaches have been advocated, such as vaccines for dogs, insecticide-impregnated collars, treatment of infected dogs, and topical insecticides, but there are still many doubts about their effectiveness.

Among these, deltamethrin-impregnated dog collars (DMC) have stood out as an available and potentially helpful tool in HVL control. John R. David and Robert Killick-Kendrick contributed significantly to the knowledge about the potential benefits of DMC as a prevention tool for HVL [6,7]. An experimental study showed that the implicated mechanism of action of DMC included both an antifeeding effect and insecticidal action that may last for up to eight months [6]. In a field trial in Brazil, they found that the seroconversion rates among dogs living in an area where collars were delivered decreased by 48% compared to culling seropositive dogs. The DMC effects on blood-feeding inhibition and increasing sand fly mortality reduce the contact of the disease vector with dogs, significant drivers of transmission [6,8,9,10].

Several studies evaluating the effectiveness of DMC use have shown satisfactory results. Field trials in endemic areas of leishmaniasis in Italy [11] and Iran [12], for example, demonstrated that dogs wearing DMC during the transmission season had a significantly lower risk of infection with *L. infantum*. The study in Iran provided the first solid evidence that a population-based intervention based on DMC could lead to a significant reduction of about 40% in the incidence rate of *L. infantum* in children [12].

In Brazil, a series of local studies also indicated the effectiveness of HVL control strategies based on the use of DMC. In Andradina/SP, for example, a study showed that DMC was associated with a reduction in canine incidence sustained for at least one year after discontinuing DMC use [13]. A field study in Capitão Enéas/MG showed that collars reduced the chances of dogs increasing antibody titers by 50% [14]. An intervention study in Governador Valadares/MG also revealed an effectiveness of DMC of about 50% in reducing canine infection incidence [15]. A theoretical study based on mathematical models demonstrated better performance of impregnated collars in reducing canine and human infection rates than euthanasia and canine vaccination [16].

Between 2011 and 2016, our research group evaluated the effectiveness of using 4% deltamethrin-impregnated collars (Scalibor^®^) as an additional measure to those already recommended by the surveillance and control program for HVL in Brazil. This strategy, including canine collars, was then implemented in “intervention” areas in seven municipalities with identified intense transmission in Brazil, simultaneously maintaining standard interventions in “control” areas in the same municipalities. Over 120,000 dogs were collared and examined throughout the project in the intervention and over 90,000 in the control areas [17].

According to the results obtained in this study, the use of collars was associated with a significant reduction of about 50% in the prevalence and incidence of canine infection when compared to areas where only the usual control actions had been implemented, meaning areas where no collaring had taken place (control area) [18,19]. Using collars impregnated with insecticide also reduced the number of captured sandflies [20]. These results are noteworthy when considering that they were observed even in the presence of a series of operational problems, such as a high rate of collar loss, indicating that addressing these difficulties could contribute to an increase in field effectiveness [21]. A subsequent cost-effectiveness study showed that the collar strategy was cost-effective for reducing canine leishmaniasis [17].

This article evaluates the effectiveness of 4% deltamethrin-impregnated dog collars (DMC) on human visceral leishmaniasis (HVL) incidence. We used data on human visceral leishmaniasis from two Brazilian cities collected over 13 years before and after the implementation of the above-cited collar study to assess to what extent the collars would have short- and long-term effects on reducing the incidence of human visceral leishmaniasis.

## 2. Methods

### 2.1. Study Site

The municipality of Araguaína, with a population of 171,301 inhabitants and an area of 4000 km^2^, is in the western mesoregion of the state of Tocantins, 384 km from the capital, Palmas [22]. The region has a humid tropical climate, with mixed vegetation showing savannah, riparian, and tropical forest characteristics. The average maximum temperature is 32 °C and the minimum 22 °C. The rainy season occurs between November and May and the dry period from June to October. The city is endemic to HVL and is considered one of the top priorities for the delivery of control action by the PSCVL.

The municipality of Montes Claros is situated in the northern region of Minas Gerais state, Brazil. It has a population of 414,240 residents and covers an area of 3589 km^2^. The city is located 422 km away from the capital, Belo Horizonte [23]. This region’s climate is hot and arid, with prevalent vegetation including the cerrado and caatinga areas. The average maximum temperature is 30 °C and the minimum 18 °C. The dry season occurs between May and September and the rainy period from November to March. The city is endemic to HVL and is considered an area at high risk for HVL occurrence.

### 2.2. Study Design and Procedures

This was a community intervention study in which the effect of 4% deltamethrin-impregnated collars on the incidence of human visceral leishmaniasis (HVL) was assessed by comparing two areas of each municipality. In the control areas, the preventive measures usually recommended by the Brazilian Program for Surveillance and Control of Visceral Leishmaniasis (PSCVL) were implemented (culling of infected dogs and vector control with residual insecticides). In the intervention areas, deltamethrin-impregnated collars were placed on domiciled dogs (those kept in a household with restricted access to the streets) in addition to the control measures applied in the control area. In the intervention areas, from June 2012 to January 2015, DMCs were delivered four times to the domiciled dogs, and there was no replacement between each distribution.

In Araguaína, both the intervention and control areas had approximately 75,000 inhabitants. The intervention area comprised 43 neighborhoods with a record of 304 HVL cases from 2008 to 2011. The control area, composed of 53 neighborhoods, registered 427 cases of HVL in the same period. In Montes Claros, the population estimates were 32 and 36 thousand for the intervention and control areas, respectively. The intervention area comprised 14 neighborhoods with 16 HVL cases from 2008 to 2011. The control area, composed of 17 neighborhoods, registered 14 cases of HVL in the same period.

The collar manufacturer company trained the field team in conducting the collaring process. The team, overseen by a veterinarian, visited households and sought domiciled dog owners’ permission to participate in the study. Once consent had been granted, the dog owner signed an informed consent document. The dog owners were advised to recognize signs of allergies, which might occur after putting on the collar. They were encouraged to contact the company’s customer support promptly for complimentary access to a veterinarian’s guidance. If an allergic reaction was suspected, the veterinarian could recommend removing the collar and, if needed, offer further assistance. Instances of allergic reactions were infrequent, and no severe reactions were reported.

### 2.3. Data Collection and Statistical Analysis

Cases of HVL (n = 1202) occurring from 2008 to 2020 were identified from the Brazilian Reportable Diseases Information System and georeferenced to the control and intervention areas in both cities. The HVL cases from 2008 to 2012 were considered to be occurring in the “pre-intervention” period. Those cases from 2013 to 2016 and from 2017 to 2020 were considered to be occurring in the “intervention” and “post-intervention” periods, respectively.

To evaluate the effectiveness of DMC against HVL, for each municipality, we performed a mixed-effects Poisson regression model using the number of population-years in each period (pre-intervention, intervention, and post-intervention) as the offset to calculate the relative rates (RR) of HVL and 95% confidence intervals (95%CI) comparing the changes from the pre-intervention period to the intervention and post-intervention periods in the intervention area relative to the control area. The intervention effectiveness (IE) was estimated to be (1 − RR)*100.

### 2.4. Ethics

This project was approved by the Oswaldo Cruz Foundation’s Committee on Animal Ethics (License LW-70/12) and found to be exempt from the need for ethical evaluation for human studies by the Pan American Health Organization (PAHO) Ethics Review Committee (Reference PAHO-2012-11-0024), since only unidentifiable aggregated secondary HVL data were used.

## 3. Results

**Figure 1:**
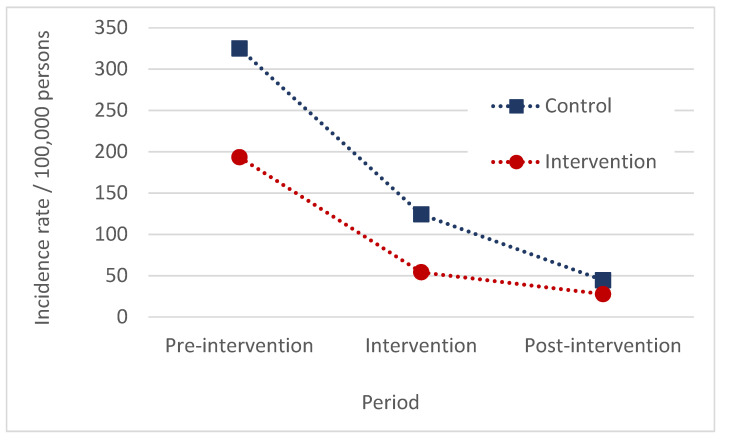
Incidence rate of HVL per 100,000 persons in the pre-intervention, intervention, and post-intervention periods in the control and intervention areas, Araguaína, Brazil, 2008–2020.

In Araguaína, there was a reduction in the incidence rate of HVL in both the control and intervention areas when comparing the intervention and post-intervention periods with the pre-intervention one (Figure 1). The average annual number of HVL cases in the intervention area was reduced from 69.6 (pre-intervention) to 19.5 (intervention period) and 10.0 (post-intervention). In the control area, the average annual number of cases was reduced from 104.0 (pre-intervention) to 39.8 (intervention period) and 14.3 (post-intervention). Comparing the intervention period with the pre-intervention period, the rate of HVL decreased by 72% in the intervention area and by 62% in the control area, yielding an effectiveness estimate of the DMC of 27% (IC95% 1–46%, *p* = 0.045). No differences were observed when comparing the pre- and post-intervention periods (*p* = 0.827) (Table 1).

**Figure 2:**
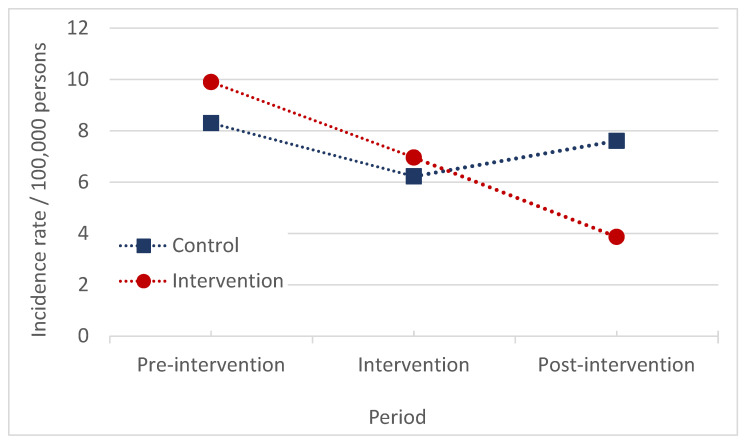
Incidence rate of HVL per 100,000 persons in the pre-intervention, intervention, and post-intervention periods in the control and intervention areas, Montes Claros, Brazil, 2008–2020.

In Montes Claros, there was a similar reduction in the incidence in both the control and intervention areas from the pre-intervention to the intervention period. In the post-intervention period, the incidence rate increased in the control area, while the incidence rate continued to decrease in the DMC area (Figure 2). The average annual number of HVL cases in the intervention area was reduced from 3.2 (pre-intervention) to 2.3 (intervention period) and 1.3 (post-intervention). In the control area, the average annual number of cases was reduced from 3.0 (pre-intervention) to 2.3 (intervention period) and then increased to 2.8 (post-intervention). No significant effects of DMC on HVL were observed (Table 1).

## 4. Discussion

In this study, the effect of a control program based on the large-scale deployment of DMC to domiciled dogs on the incidence of HVL showed conflicting results. In the city of Araguaína, the use of DMC was associated with a reduction of 27% in the incidence of HVL in the four years in which the intervention occurred compared to the pre-intervention period. However, no significant reduction associated with DMC was detected after this period, suggesting that a control program based on the large-scale deployment of DMC might have to be maintained for more extended periods without interruption.

In Montes Claros, the incidence of HVL decreased equally from the pre-intervention to the intervention period in both the control and intervention areas (*p* = 0.913). Although in the post-intervention period the incidence increased in the control area while cases continued to decrease in the DMC area, such difference was not statistically significant (*p* = 0.188), indicating that a preventive program based on DMC has no significant effect on the incidence of HVL in this area.

Such inconsistency in the results might reflect a valid variation in the putative effect of collars in different levels of transmission intensity, as occurs in field trials to evaluate vaccine effectiveness [24]. In such a situation, the effect of collars in decreasing the incidence of HVL is likely to be more feasible in areas with high transmission rates and large numbers of cases, as in Araguaína. Unfortunately, there are no available data on the seroprevalence of anti-Leishmania antibodies in representative cohorts of humans before and post intervention in the control and intervention areas to permit a more detailed and formal quantitative evaluation of such a hypothesis. However, data on canine leishmaniasis showed that there was a non-significant difference between the baseline prevalence of canine leishmaniasis in the control (10.5%) and intervention (9.7%) areas (*p* = 0.637) in the city of Montes Claros. Yet, in Araguaína, the prevalence of canine leishmaniasis in the first two years of the study was higher in the control (26.1%) than in the intervention (11.2%) areas (*p* < 0.001).

It is worth noting that the incidence of HVL decreased from the pre-intervention (2008–2012) to the intervention period (2013–2016) in both the control and intervention areas in the two studied cities, suggesting that some structural modifications in healthcare and socioeconomic conditions may have contributed for such a generalized drop in the incidence. However, the interval from 2008 to 2016 is relatively short for detecting significant social changes that influenced the dynamics of HVL. Indeed, a composite index of socioeconomic development, evaluating income, education, and health, remained relatively the same in both cities from 2008 to 2016 [25]. Alternatively, improvements in the local control programs for visceral leishmaniasis might have contributed to the observed decreased trend in HVL incidence in both cities. However, no data are available to check such a hypothesis. Finally, such a decreasing trend might reflect a cyclical pattern of leishmaniasis transmission, as observed in different regions of Brazil [26].

The low number of HVL cases in the city of Montes Claros might have affected the statistical power of the study to detect small differences between the control and intervention areas as being statistically significant [27]. Additionally, socioeconomic and environmental heterogeneities influence the intensity and seasonality of transmission in each city, likely contributing to the conflicting results [28]. The eventual differences in the level of implementation of other control strategies among cities may also be an unmeasured confounding factor [28]. Differential rates of collar loss between cities could also influence the degree of effectiveness of the DMC control program [29]. The varying dynamics of the dog population also affect the effectiveness of DMC. For instance, the fast turnover of the dog population may make maintaining high levels of collar coverage challenging, and the consequent introduction of newly susceptible animals contributes to sustained transmission [18]. Finally, spillover effects between units might affect the validity of effectiveness estimates of intervention [30,31]. For instance, the repellent effect of DMCs in an intervention area might increase the sand fly population in a control area [32].

Whatever the limitations, this study has some originalities and strengths. First, a few community intervention trials have evaluated the effectiveness of DMC in reducing HVL cases [12], and all the others have examined canine leishmaniasis [33]. Human studies are critical because the effectiveness of DMC in reducing infection in dogs may not guarantee that a similar effect would be seen regarding human VL [34]. Another positive feature of this study was its long-term evaluation, including measures of HVL rates up to four years after the intervention had finished. Although variations between cities can potentially bias the results, the differences in effectiveness observed might well represent plausible causal mechanisms, reinforcing that a one-for-all solution is probably inadequate, at least in the visceral leishmaniasis context, in which the local landscape is likely to be of great importance [35].

The divergent results obtained from Araguaína and Montes Claros suggest that DMC could be used as an additional strategy for controlling visceral leishmaniasis in Brazil in areas with a high transmission levels and numbers of cases, but it needs to be sustained. Our cost-effectiveness analysis showed that DMCs are cost-effective means of preventing canine leishmaniasis [17]. However, since the effectiveness estimate for preventing HVL is relatively small and DMCs are expensive, further cost-effectiveness analyses should be performed to support introducing such preventive devices as a large-scale control measure for HVL.

## 5. Conclusions

The use of DMC was associated with a reduction of 27% in the incidence of HVL during the period of DMC delivery, indicating that DMC is effective as an additional strategy for controlling visceral leishmaniasis in Brazil. However, no significant reduction associated with DMC was detected after the intervention period, suggesting that a control program based on the large-scale deployment of DMC might have to be maintained for more extended periods without interruption.

## Figures and Tables

**Table 1:** Relative risk of HVL associated with DMC intervention and DMC effectiveness comparing the incidence of HVL in the intervention and post-intervention periods with the pre-intervention incidence, Araguaína and Montes Claros, Brazil, 2008-2020.

City/Area	Intervention Period vs. Pre-Intervention			Post-Intervention Period vs. Pre-Intervention		
	RR (95%CI)	IE (%) (95%CI)	*p*-Value	RR (95%CI)	IE (%) (95%CI)	*p*-Value
Araguaína						
Control	1			1		
Intervention	0.73 (0.54–0.99)	27 (1–46)	0.045	1.04 (0.68–1.60)	−4 (−60–32)	0.827
Montes Claros						
Control	1			1		
Intervention	0.93 (0.29–2.99)	7 (−100–71)	0.913	0.42 (0.11–1.51)	58 (−49–89)	0.188

## Data Availability

All relevant data are within the paper.
